# A Novel Approach to Repair of Tracheal Occlusion Secondary to Percutaneous Tracheostomy Creation

**DOI:** 10.7759/cureus.33868

**Published:** 2023-01-17

**Authors:** Ali Syed, Mohammed A Kamalia, Alison Messina, Shahriyour Andaz, Joshua Melamed, Vanessa Gibson

**Affiliations:** 1 Medicine, Medical College of Wisconsin, Milwaukee, USA; 2 Vascular Surgery, Temple University Hospital, Temple, USA; 3 Thoracic Surgery, Mount Sinai South Nassau, Oceanside, USA; 4 Thoracic Surgery, Medical College of Wisconsin, Milwaukee, USA

**Keywords:** tracheal obstruction, nd:yag laser, sub-laryngeal occlusion, central airway obstruction, airway obstruction

## Abstract

The number of endotracheal intubations increased in the United States during the COVID-19 pandemic with an associated rise in laryngotracheal injury. Our patient had a complete laryngeal occlusion just proximal to the first tracheal ring. The Neodymium-doped Yttrium Aluminum Garnet (Nd-YAG) laser is often used to resolve sub-laryngeal occlusions, and without access to the Nd-YAG laser, we had to find an alternative solution. Few centers have the access to an Nd-YAG laser, the optimal choice for sub-laryngeal occlusion and our novel approach allowed us to reestablish tracheal continuity and the patient’s ability to speak.

## Introduction

The number of endotracheal intubations increased in the United States during the COVID-19 pandemic with an associated rise in laryngotracheal injury [1,2]. We discuss a patient who developed tracheal occlusion after undergoing percutaneous tracheostomy creation for COVID-19-associated respiratory failure. A novel minimally invasive approach using a percutaneous tracheostomy kit in a retrograde fashion was utilized to perform multiple dilations through the existing tracheostomy. The patient had an uneventful perioperative and postoperative course and could speak clearly for the first time in 5 months at the end of our case. Few centers have the access to a Neodymium-doped Yttrium Aluminum Garnet (Nd-YAG) laser, the optimal choice for sub-laryngeal occlusion and our novel approach allowed us to reestablish tracheal continuity and the patient’s ability to speak. Here we detail our approach, which can be adopted to improve patient outcomes at secondary hospitals.

## Case presentation

A 65-year-old Hispanic woman was intubated for COVID-19-associated respiratory failure at the height of the crisis in March 2020. She underwent percutaneous tracheostomy creation at an outside hospital and was eventually discharged. She was admitted to us approximately 10 weeks after her previous discharge, after an acute tonic-clonic seizure episode. A neurologic workup was negative for seizure activity. She was unable to tolerate brief periods of tracheostomy capping, concerning for tracheal stenosis. Bronchoscopy through the oropharynx demonstrated complete laryngeal occlusion just proximal to the first tracheal ring. Completion bronchoscopy performed through the tracheostomy tube did not demonstrate distal airway pathology. Esophagoscopy showed no evidence of tracheoesophageal fistulae.

**Figure 1:**
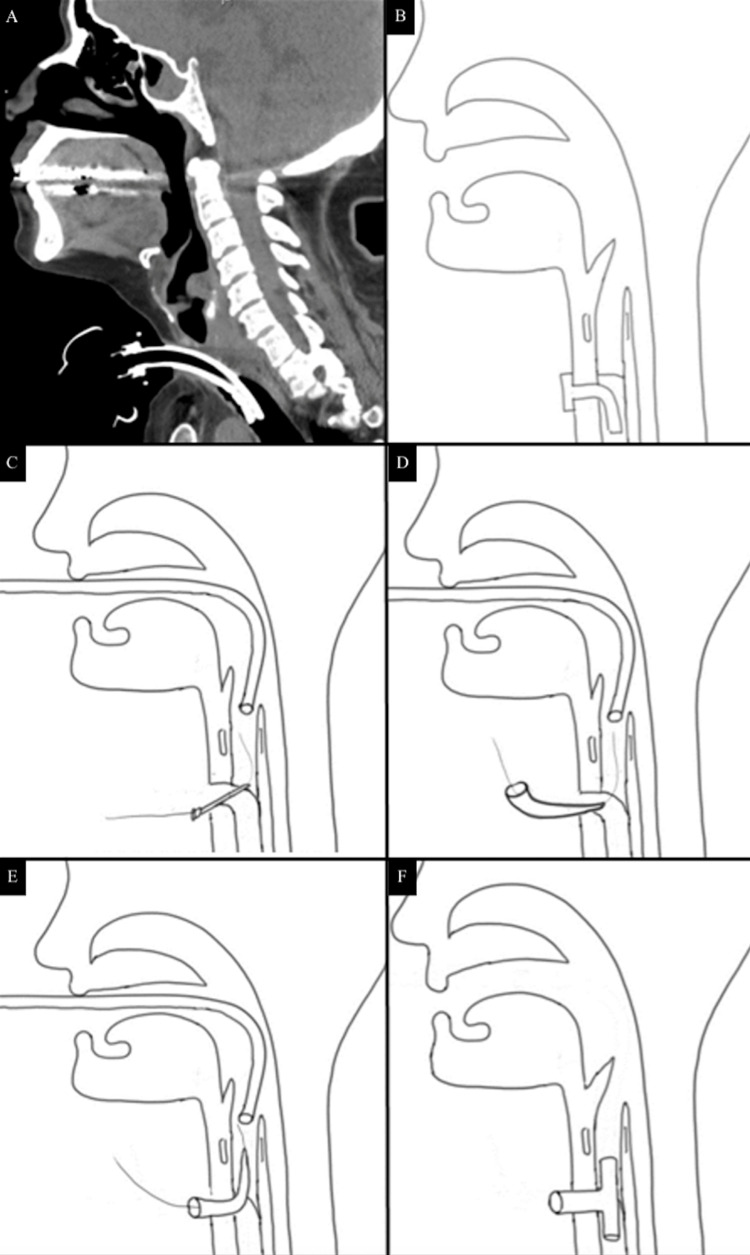
Computerized Tomography of Occlusion and Visualization of Novel Repair Technique A) Computed tomography imaging revealing tracheal obstruction proximal to the tracheostomy site. B-F) Original illustration of retrograde dilation technique through existing tracheostomy site and subsequent stenting with a Montgomery® Safe-T-Tube™.

Laryngeal occlusion can be managed in multiple ways. Therapy for laryngeal occlusion is dichotomized into two arms between life-threatening obstructive disease and non-life-threatening disease. Given that our patient already had a patent tracheostomy tube we were left to decide amongst the various non-life-threatening disease management options. There are a wide variety of options such as bronchodilator use for exercise-induced laryngeal obstruction, bronchoscopic ablation/cryotherapy for hypercontraction of the vocal cords to supraglottic laryngotomy for malignant obstruction, or critical stenosis [3,4]. At this point, we referenced a technique commonly used for tracheal obstruction, the Nd-YAG laser. The Nd-YAG laser is more commonly used in ocular surgery procedures, however, in cases of airway obstruction either due to malignant obstruction or emergent airway obstruction it can also be used in thoracic and ENT procedures. The laser often proves most useful in patients with malignant lesions that are obstructing, often with little vocal cord damage or post-laser adhesions [5]. Due to the low complication rate, and safe approach demonstrated by the Nd-YAG laser, it would likely have been our first choice. However, at our institution, we did not have access to a laser. Given our unique situation, we thought of a novel approach to treating our patient, which we have detailed below in Figure 1.

We opted to repair our patient’s occlusion using a Cook Ciaglia Blue Rhino® tracheostomy kit, and here we describe our step-by-step approach. First, the patient was laid supine on the operating room table, anesthetized and LMA® Protector™ Airway with Cuff Pilot™ was inserted. The patient was oxygenated through her existing tracheostomy site with FiO2 of 100% for 5 minutes. Second, a bronchoscopy was then inserted down to the point of occlusion. The patient was decannulated and the needle from our Cook Ciaglia Blue Rhino® tracheostomy kit was inserted retrograde through the point of occlusion until it was visualized by the bronchoscope. Care was taken to pass the needle anteriorly so as to avoid the membranous portion of the trachea and potential esophageal injury (Figure 1C). Third, a wire was passed through the needle and the needle was removed. At this point using the Seldinger technique the tract was sequentially dilated using the dilators up to 26 Fr (Figure 1E). Fourth, the patient was recannulated and placed back on the ventilator to be reoxygenated. Using a bronchoscope, the distance from a few millimeters below the vocal cords was measured down to the tracheostomy. Using that measurement, a Montgomery® Safe-T-Tube™ was cut to size. Fifth, once the patient was saturating at 100%, she was once again decannulated. The Montgomery® Safe-T-Tube™ was folded on itself and inserted into the tracheostomy traversing the previous area of occlusion. The ventilator was then connected to the LMA® Protector™ Airway with Cuff Pilot™(Figure 1F). Sixth, a completion bronchoscopy was performed through the Montgomery® Safe-T-Tube™ to prove patency and ensure that the superior portion was not abutting the vocal cords.

Immediately following surgery in the post-anesthesia care unit, our patient was able to speak clearly after 5 months of not being able to do so.

## Discussion

In today’s literature, there are very few options for sub-laryngeal occlusion, we report one of the first cases regarding a patient with COVID-19-induced pneumonia. Most patients afflicted with severe acute respiratory syndrome coronavirus 2 (SARS-CoV-2) successfully recover with supportive care, but 10-15% of hospitalized patients require prolonged ventilatory support, which may lead to laryngotracheal injury [1]. Although rare, patients who undergo prolonged mechanical ventilation during treatment for COVID-19 can develop chronic airway problems or post-intubation tracheal stenosis [6]. These patients developed signs of airway problems several weeks following extubation and recovery. One study concluded the incidence of tracheal complications following invasive mechanical ventilation, including full-thickness tracheal lesions, and tracheoesophageal fistulas, to be more frequent in patients with COVID-19 in comparison to matched controls [7]. Patients with post-intubation tracheal stenosis or occlusion are often referred for tracheal resection, but this may not be ideal in frail patients or centers without expertise in tracheal surgery. The first case report of a patient who developed laryngotracheal stenosis following intubation and tracheostomy due to COVID-19 pneumonia was managed with microlaryngoscopy, laser excision, and balloon dilation [8]. In the last month, we have seen a case series regarding a bronchoscopic retrograde recanalization approach of a similar set of pathologies, this shows that our novel approach has the potential to become pervasive [9]. This utilization of our technique may prove beneficial for surgeons who may not have access to advanced endoscopic instruments or have patients that are too frail to undergo extensive tracheal resections.

## Conclusions

The COVID-19 pandemic has increased the number of patients requiring prolonged invasive ventilatory support, thereby increasing the potential for subsequent laryngotracheal injury. In patients who had previously undergone tracheostomy, subglottic occlusion may be successfully treated with dilation through the tracheostomy and reinforcement around a T-tube. This novel approach is simple, cost-effective, and safe and it increases the accessibility of surgical procedures to centers without extensive equipment. Although it may only be a temporizing measure for patients that may eventually require tracheal resection, it provides short-term relief and palliation for patients who may not tolerate more extensive surgery.
